# Lignin–Inorganic Interfaces: Chemistry and Applications from Adsorbents to Catalysts and Energy Storage Materials

**DOI:** 10.1002/cssc.202000216

**Published:** 2020-04-17

**Authors:** Tetyana M. Budnyak, Adam Slabon, Mika H. Sipponen

**Affiliations:** ^1^ Department of Materials and Environmental Chemistry Stockholm University Svante Arrhenius väg 16C SE-106 91 Stockholm Sweden

**Keywords:** interfaces, lignin, nanoparticles, organic–inorganic hybrid composites, sustainable chemistry

## Abstract

Lignin is one the most fascinating natural polymers due to its complex aromatic‐aliphatic structure. Phenolic hydroxyl and carboxyl groups along with other functional groups provide technical lignins with reactivity and amphiphilic character. Many different lignins have been used as functional agents to facilitate the synthesis and stabilization of inorganic materials. Herein, the use of lignin in the synthesis and chemistry of inorganic materials in selected applications with relevance to sustainable energy and environmental fields is reviewed. In essence, the combination of lignin and inorganic materials creates an interface between soft and hard materials. In many cases it is either this interface or the external lignin surface that provides functionality to the hybrid and composite materials. This Minireview closes with an overview on future directions for this research field that bridges inorganic and lignin materials for a more sustainable future.

## Graphical abstract

**Lignin–inorganic hybrid materials**: Lignin can provide reactive phenolic interfaces to inorganic materials. It is often this interface or external surface that brings synergic effects and completely new functionality useful in applications. This Minireview takes a critical look at the most intriguing examples of lignin–inorganic hybrids, composites, and other materials from the viewpoint of materials chemistry.

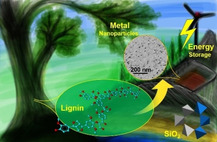

## 1 Introduction

**Figure 1:**
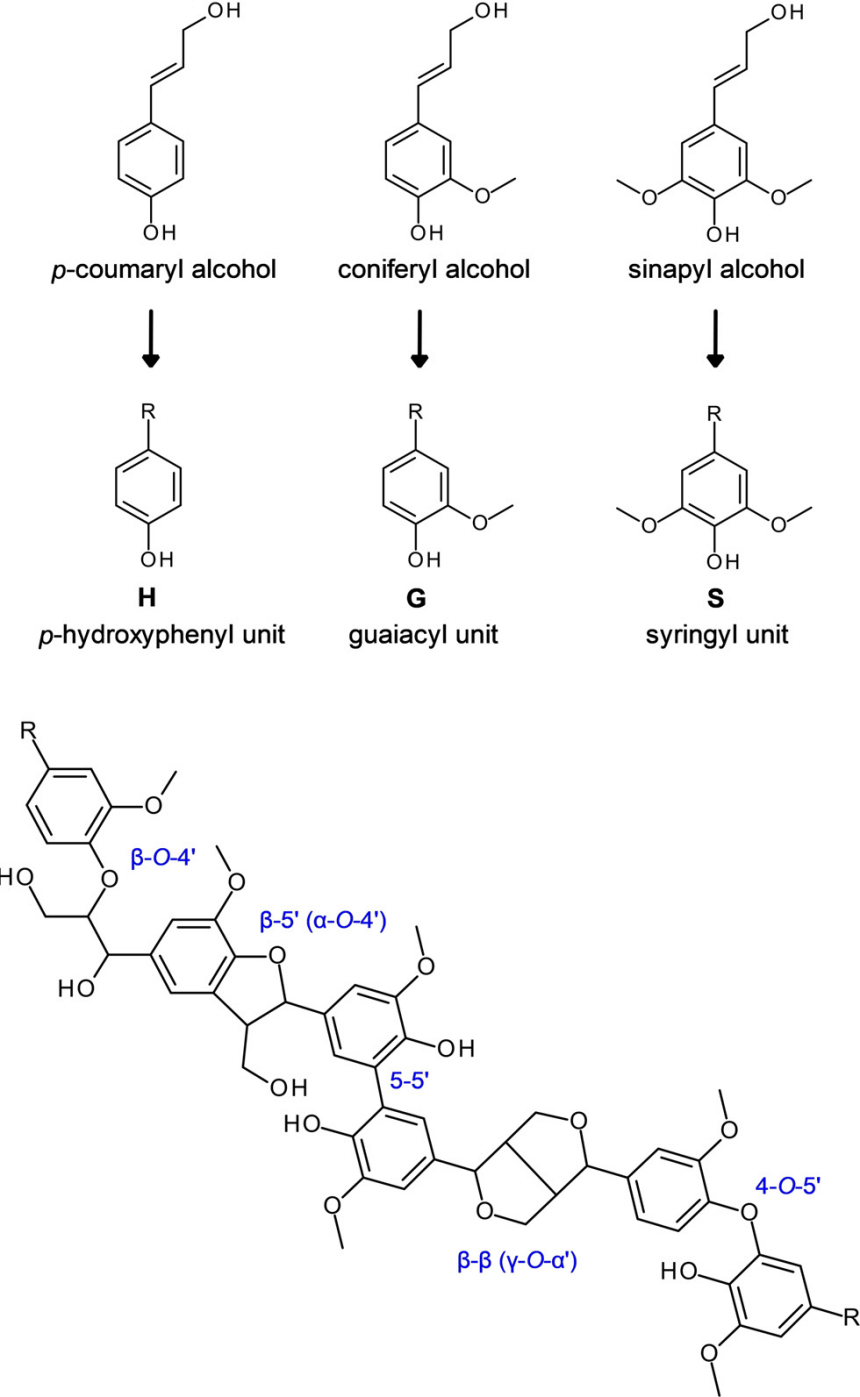
Monomeric precursors of lignin, H, G, and S structural units, and a lignin structure model showing five guaiacyl units connected by five of the most common interunit linkages. R=lignin.

Polyphenolic lignin reinforces plant cell walls and provides barrier properties against water, free radicals, microbes, and insects.^1^, ^2^, ^3^, ^4^, ^5^ Three main monomeric precursors give rise to lignin oligomers/polymers and their *p*‐hydroxyphenyl (H), guaiacyl (G), and syringyl (S) structural units (Figure 1). Structural diversity of lignin is considerable between and within different plant species, and it is generally considered that the likelihood of finding two identical lignin macromolecules is negligible. Furthermore, the chemical structure and frequency of the common interunit linkages depend on the process used for isolating lignin from wood.^6^, ^7^

Lignin is often cited as an abundant but underutilized natural polymer.^8^, ^9^ As a starting material, lignin can be derived from low‐cost sidestreams of existing industrial processes,^10^, ^11^, ^12^ and it is thus a promising sustainable resource, provided that the principles of green chemistry are followed.^13^ The quantity of lignin fixed in forest biomass is indeed enormous, as it has been estimated that there are three trillion trees on Earth.^14^ From data on aboveground forest biomass,^15^ the global amount of lignin can be estimated at 10^11^ metric tons, if an average lignin content of 25 % is assumed. The pulp and paper industry is the primary source of lignin in isolated form. About 50–70 million tons of lignin is annually dissolved from wood, but only 1–2 % of this total amount is isolated, while the majority is combusted to recover the inorganic cooking chemicals.^16^

One of the obstacles that have hindered the supply of lignin is the lack of commercial applications in a sufficiently high total volume. Structural complexity of lignin is one of the main challenges for its conversion to chemicals^17^ or valorization in materials.^18^ Recent years have seen increasing interest in lignin‐based materials such as lignin nanoparticles (LNPs),^19^, ^20^, ^21^, ^22^, ^23^, ^24^ which are expected to enable the use of lignin in value‐added applications.^25^, ^26^ Owing to the abundant phenolic hydroxyl groups, lignins exhibit antioxidant and UV‐absorbing properties, which have been harnessed in polymer blends, composites, and thermoplastics,^27^ as reviewed recently.^28^, ^29^ The lignin research field is becoming increasingly interdisciplinary. In addition to its use in organic polymer matrices, several properties of lignin, such as amphiphilicity, surface activity, and redox reactivity, are valuable for materials containing inorganic interfaces.

**Figure 2:**
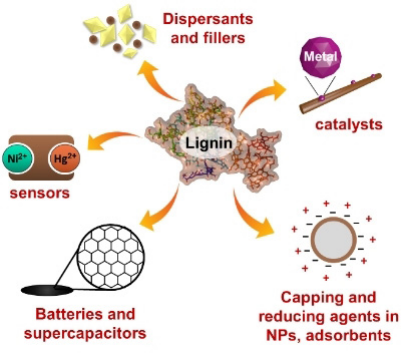
Application areas of lignin in inorganic material interfaces covered in the present Minireview.

There are many instances in which lignin facilitates either the synthesis or functionalization of an inorganic material (Figure 2). The purpose of this Minireview is to critically discuss the materials chemistry basis for the use of lignin in applications in which inorganic components play a central role. Besides lignin–inorganic composite materials, this also includes lignin–inorganic interfaces that can be formed in situ under operating conditions and trigger the target application. As inorganic materials, all materials of inorganic origin, from inorganic oxides to metal ions, will be considered. In addition, lignin‐derived carbonaceous materials are appreciated as components of energy storage materials. The focus of our Review is on benefits brought by lignin to the synthesis and application of selected materials such as adsorbents, batteries, catalysts, dispersants, and sensors (Figure 2). We close with our perspectives and suggestions for the most promising future horizons for lignin in materials containing inorganic interfaces.

## 2 Materials and Applications

### 2.1 Adsorbents

Lignins are characterized by aromatic motifs carrying phenolic hydroxyl groups and a side chain containing aliphatic hydroxyl and negatively charged groups (e.g., carboxyl and/or sulfo, depending on the pulping process). It therefore seems advantageous to combine “soft” lignin with “hard” inorganic materials that lack these organic functionalities. Inorganic materials as such are effective adsorbents for different contaminants in aqueous solutions, for example, metal ions and organic pollutants,^30^, ^31^ and are characterized by a rigid structure, high thermal stability and specific surface area, as well as resistance to microbial attack. The combination of lignin with inorganic adsorbents is also motivated by modification of the specificity, capacity, kinetics, and pH range of adsorption. Several strategies have been used to synthesize lignin–inorganic composites: sol–gel method,^32^, ^33^ adsorption with modified^34^, ^35^ or unmodified^36^ lignin, co‐precipitation,^37^ and so forth. Since the chemical nature of the surface defines the type of interaction with an adsorbate,^38^ variations in the source and properties of lignin influence the adsorption process.^39^ Table 1 summarizes adsorption capacities of lignin–inorganic composites to organic molecules and metal ions. Although a direct comparison of adsorption capacity is not always possible because of the different pH values and temperatures used, some trends are apparent.

**Table 1:** Lignin–inorganic hybrid materials and their performance as adsorbents.

Material	Adsorbate	Max. adsorption capacity [mg g^−1^|mg m^−2^]	Ref.
hydrolyzed lignin/SiO_2_	2,4‐dichloro‐phenoxyacetic acid	4.9|0.03	32
oxidized lignoboost kraft lignin/SiO_2_	crystal violet	110.1|1.3	35
kraft lignoboost lignin/SiO_2_	methylene blue	41.6|0.6	49
fractionated kraft lignin/SiO_2_	methylene blue	60.0|0.7	49
lignosulfonate/Fe_3_O_4_ microspheres	methylene blue	283.6|6.3	48
alkali lignin‐dopamine/Fe_3_O_4_ NPs	Cr^3+^	44.6	50
carboxymethylated lignin–Fe_3_O_4_/SiO_2_	Cu^2+^ Pb^2+^	70.7 150.3	51
wheat straw lignin/montmorillonite hydrogel	Cu^2+^	74.4	52
lignin/MgO–SiO_2_	Cu^2+^	83.9|0.39	53
lignin/MgO–TiO_2_	Cu^2+^	36.0|0.12	54
	Cd^2+^	69.8|0.22	
lignin/SiO_2_–TiO_2_	Cu^2+^	19.2|0.22	54
	Cd^2+^	16.7|0.19	
lignin/TiO_2_	Cu^2+^	20.1|0.5	54
	Cd^2+^	22.4|0.57	
lignosulfonate–graphene oxide hydrogel	Pb^2+^	1308|2.8	55

Like intrinsically anionic lignin adsorbents,^40^, ^41^, ^42^, ^43^, ^44^ lignin–inorganic composites have been successfully applied for adsorption of cationic dyes. Methylene blue and crystal violet dyes were removed by oxidized or amine‐functionalized fractionated kraft lignin–silica composites with 10–50 % higher capacity compared with the initial pure lignins and 20–90 % higher capacity compared with the silica material.^33^, ^35^ Simple recalculation of composite adsorption capacity to square meters of silica support showed that the composites remove dyes at the level of activated carbon (0.24–0.64 mg m^−2^).^45^, ^46^, ^47^ Wang and co‐workers reported an even higher uptake of 283.6 mg g^−1^ or 6.3 mg m^−2^ of methylene blue on lignosulfonate–Fe_3_O_4_ core–shell particles.^48^ The studied effects in the aforementioned works point to the fact that the nature of support and amount of lignin on the surface play significant roles in the adsorption properties of the lignin–inorganic adsorbents.

In some cases, adsorption of heavy‐metal cations on lignin–inorganic hybrid materials is more efficient than that reported for many different types of lignins. For instance, lignin/MgO–SiO_2_ and lignin/MgO–TiO_2_ exhibited adsorption capacities of 84 and 70 mg g^−1^ for Cu^2+^ and Cd^2+^,^53^, ^54^ whereas lignin (Cu^2+^ 1.7–26 mg g^−1^, Cd^2+^ 6.7–48 mg g^−1^)^56^ and activated carbon (Cu^2+^ 9–38 mg g^−1^, Cd^2+^ 3.7–146 mg g^−1^)^57^ showed lower values. However, considerably higher adsorption capacities for Cu^2+^ (87 mg g^−1^) and Cd^2+^ (137 mg g^−1^) have been reported for lignin precipitated from black liquor originating from pulping of Eucalyptus wood.^58^ Differences in the source and cooking parameters have been found to affect the intrinsic adsorption properties of kraft lignins.^59^ Morevoer, chemical modification of lignin may improve its adsorption capacity, provided that the accessibility of the adsorption sites is not decreased. Dai and co‐workers used EDC/NHS chemistry to synthesize dopamine–alkali lignin amides that were afterwards nanoprecipitated on Fe_3_O_4_ NPs.^50^ The adsorption capacity of the hybrid particles for Cr^3+^ was 44.6 mg g^−1^, which is higher than those of LNPs or many other organic materials.

**Figure 3:**
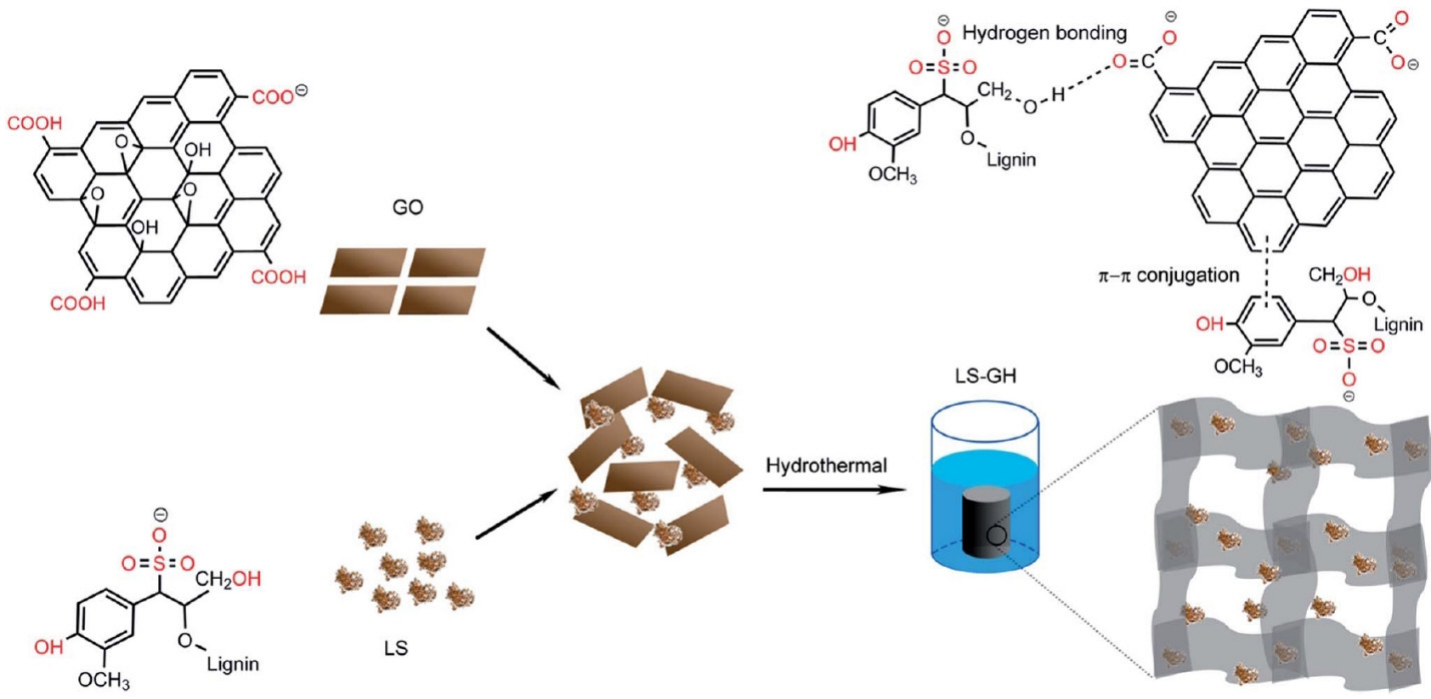
Synthesis of one‐step self‐assembled lignin–graphene and noncovalent interactions between reduced graphene oxide sheets and lignin chains. Reproduced from Ref. 55 with permission from The Royal Society of Chemistry.

Hydrogels are promising materials for water purification, since they are characterized by high surface areas and adsorption capacities combined with fast kinetics. Li and co‐workers synthesized a lignosulfonate–graphene hydrogel that exhibited an adsorption capacity of 1308 mg g^−1^ of Pb^2+^ over 40 min (Figure 3).^55^ The hydrogel showed good regeneration performance during ten adsorption–desorption cycles. Despite all the above‐discussed results, the literature is nonetheless lacking discussion on the stability of lignin‐based adsorbents, not only in terms of repeated use of the material, but also regarding resistance against leaching of lignin into the treated water.

### 2.2 Antimicrobial materials

Antimicrobial activity of a variety of different lignin fractions has been reported,^60^, ^61^, ^62^, ^63^, ^64^, ^65^, ^66^ but isolated lignin cannot be regarded as antimicrobial without experimental validation. Nevertheless, lignin is a poor carbon source for microbes and, in turn, well‐suited as a component of antimicrobial materials.^67^ The most common strategies include incorporation of silver ions or NPs in lignin‐based organic^68^ and inorganic^69^ composite materials, and infusion of silver ions into LNPs.^70^, ^71^ Lignin also functions as a reducing agent in the preparation of silver NPs^72^, ^73^, ^74^, ^75^, ^76^ and composite materials with antioxidant activity.^74^, ^77^, ^78^ Phenolic hydroxyl groups are the active species that undergo oxidation to quinones and release one hydrogen ion and one electron, which reduces Ag^+^ to Ag^0^. It has also been found that fragmentation of the aliphatic side chain of lignin occurred when the formation of reactive quinone methide species took place under microwave irradiation.^79^ The use of lignin in redox processes requires therefore delicate control over such fragmentation reactions.

Harnessing the redox properties of lignin, Gan et al. prepared a mussel adhesive‐mimicking material based on silver–lignin hybrid nanoparticles entrapped in poly(acrylic acid) hydrogel.^29^ Lignin had two distinct functions in this material. First, lignin reduced silver ions in the hybrid particles. Second, deprotonation of acrylic acid generated a proton flux that, together with the electrons arising from silver NPs, formed a dynamic redox system in which catechol groups were in equilibrium with the quinones. The resulting ductile hydrogel showed strong adhesion on various substrates, including steel, glass, synthetic polymer, and biological tissues.

### 2.3 Dispersants and fillers

Dispersing of inorganic materials is an existing high‐volume application for lignin. Due to their amphiphilic structures and anionic surface charge, lignosulfonates and other lignin‐based materials have been used as dispersants and plasticizers for graphene,^80^ kaolin,^81^, ^82^ alumina,^83^ cement,^84^, ^85^, ^86^ and concrete^87^, ^88^ suspensions. For example, in the case of alumina (Al_2_O_3_), the use of lignosulfonates facilitates reaching sufficiently high concentrations of inorganic particles that are required in colloidal production of ceramic materials.^83^ In cement, lignosulfonates accelerate setting,^85^ increase strength,^86^ and inhibit the hydration reaction, as indicated by a lower degree of silicate polymerization.^84^ When dispersed in graphene, lignin provided anticorrosion properties to an epoxy coating prepared from the nanocomposite.^80^ Structural hydroxyapatite–lignin composites have been reported as functional materials for bone tissue osseointegration^89^ and antifouling films doped with silver ions.^90^ Lignin has also been studied as a binder to stabilize silt for mechanically improved properties of foundation soil for construction purposes.^91^

Amorphous silica and lignin share some common properties, such as lack of crystal structure and solubility in aqueous alkali (e.g., in soda pulping of cereal straw),^92^ which makes their composites and copolymers lucrative as fillers for synthetic^93^, ^94^, ^95^, ^96^, ^97^, ^98^, ^99^ and natural fiber^100^ polymer matrices. Chemically modified siloxane–lignin–polymer materials have been proposed for electroactive blends,^101^ anticorrosion coatings,^102^ and antioxidant additives.^92^ Furthermore, Hayashi et al. used lignin to modify the pore size distribution of silica.^103^ They reported that a lignin/silica weight ratio of 0.01 was sufficient to reduce the pore size from approximately 8 nm to approximately 1 nm, and at a weight ratio of 0.1 the pore size was below the analytical threshold. Possible benefits of this porosity control by lignin remain to be demonstrated, but could include, for instance, pH‐responsive drug release.

### 2.4 Sensors for metal ions and hydrogen peroxide

**Figure 4:**
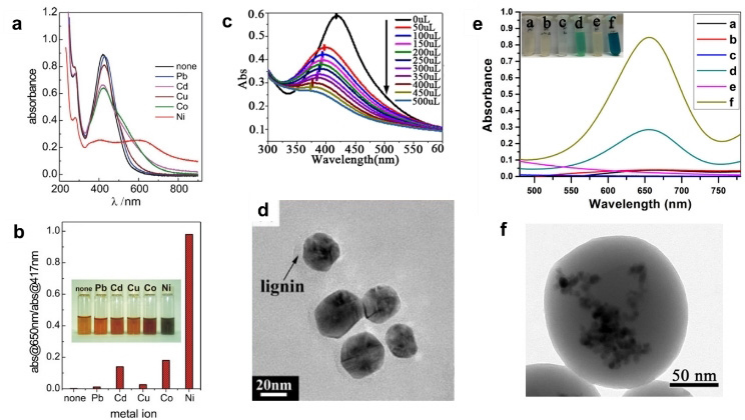
Examples of lignin–inorganic nanomaterials as sensors for a) Ni^2+^ ions by lignin‐capped silver NPs, observed in UV/Vis absorption spectra in the presence and absence of various metal ions, and b) the metal‐ion sensing capability of the lignin–Ag NPs expressed as the change in absorption ratio at wavelengths of 650 and 417 nm. Reproduced with permission from ref. 104; Copyright Elsevier. c) Hg^2+^ ions by lignin‐capped silver NPs and d) a TEM image of the particles. Reprinted with permission from Ref. 79; Copyright American Chemical Society. e) UV/Vis spectra of a) LNPs+3,3′,5,5′‐tetramethylbenzidine (TMB), b) LNPs+TMB+H_2_O_2,_ c) Fe_3_O_4_+TMB, d) Fe_3_O_4_+TMB+H_2_O_2_, e) Fe_3_O_4_ entrapped in LNPs+TMB and f) Fe_3_O_4_ entrapped in LNPs+TMB+H_2_O_2_; f) HRTEM image of individual Fe_3_O_4_ NPs entrapped in an LNP. Reproduced from Ref. 106.

There are a few interesting examples of lignin in sensor applications. Figure 4 a and b show the work of Milczarek et al., who used lignosulfonate as a reducing and capping agent in the synthesis of Ag NPs for colorimetric sensing of Ni^2+^ ions (detection limit 20 μm).^104^ The decrease in absorbance of metallic silver at 432 nm has been utilized in several metal sensors. Figure 4 c and d show a similar approach using lignin from acetic acid pulping for the synthesis of lignin‐capped Ag NPs for sensing of Hg^2+^ ions.^79^ Although sensing of nickel ions was not studied for comparison, the system was markedly sensitive (detection limit 23 nm) and selective to Hg^2+^ without major interference from the presence of other tested ions. A few groups have investigated lignin‐based hybrid particles for the sensing of hydrogen peroxide.^105^, ^106^, ^107^ These methods rely on oxidation of 3,3′,5,5′‐tetramethylbenzidine (Figure 4 e and f)^106^ or metallic silver^105^, ^107^ in the presence of H_2_O_2_. These colorimetric methods require such low concentrations of NPs that the absorbance spectrum of lignin does not appear to cause notable interference. A sensor material containing hierarchical zinc oxide (ZnO) nanorods grown in the presence of lignin was used for sensing of ammonia gas based on the semiconducting properties of ZnO.^108^ However, ZnO nanostructures have been known for decades for gas sensing applications.^109^, ^110^

### 2.5 Materials for heterogeneous catalysis and photocatalysis

The petrochemical and pharmaceutical industries rely on heterogeneous catalysts, and a major challenge is the transformation of established catalytic processes towards sustainable solutions.^13^ This applies also for homogeneous catalysts, which can exhibit high mass activity, but their subsequent recycling is tedious and very often even not possible, for example, in catalytic oligomerization reactions.^111^ Likewise to the synthesis of silver NPs for antimicrobial activity, sensors,^104^ and catalysis,^112^ lignin has been used as a complexing, reducing, and capping agent for the synthesis and stabilization of gold, palladium, ruthenium, and rhenium^113^, ^114^, ^115^, ^116^, ^117^ NPs. Lignin‐capped palladium NPs were used to catalyze cross‐coupling reactions in water with good selectivities, indicating that the presence of lignin does not impede catalytic activity of metal NPs.^116^ The ability of lignin to stabilize metal NP dispersions is related to the polyelectrolyte layer formed on the surface of metal NPs.^117^, ^118^ However, it is not clear which are the most suitable charged groups, net surface charge, and molecular weight distribution of lignin in this application.

Lignin represents an alternative to silica, alumina, and carbon as support materials for metal catalysts. Nasrollahzadeh et al. prepared magnetic calcium lignosulfonate‐supported Pd complexes for the aqueous Suzuki–Miyaura C−C coupling reaction.^119^ However, note that the Suzuki coupling reaction can be already catalyzed by trace amounts of Pd, and it is therefore probably not the ideal reaction for benchmarking lignin‐supported metal catalysts.^120^ Another example is the Huisgen [3+2] cycloaddition on cuprous oxide (Cu_2_O) nanoparticle catalysts deposited on spherical LNPs obtained from alkali lignin.^121^ Moreover, anchoring cobalt NPs on carbon obtained from lignin pyrolysis under carbon dioxide atmosphere yields a composite catalyst for the activation of the peroxomonosulfate (oxone) ion.^122^ Since the presence of magnetic NPs facilitates catalyst separation, this is another example of green chemistry covering both catalyst preparation and recycling.

**Figure 5:**
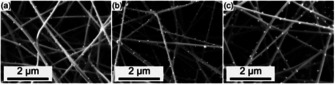
Lignin‐derived conductive electrospun carbon nanofibers containing surface‐bound Ag NPs at Ag loadings of a) 11 wt %, b) 15 wt %, and c) 25 wt %. Reproduced with permission from Ref. 127; Copyright Elsevier.

Besides the above‐mentioned thermocatalytic applications, lignin is also a promising precursor to synthesize carbon‐supported metal NPs that can be used as heterogeneous catalysts for electrochemical reactions. Electrocatalysis is considered as one of the key green technologies for hydrogen generation due to mild reaction conditions including temperature and pressure.^123^ Direct alcohol fuel cells use hydrogen generated from the electrochemical oxidation of C_1_ or C_2_ alcohols.^124^ Carbonization of lignin derived from black liquor and subsequent acidic treatment was reported to give carbon dots with a large number of oxygen‐containing groups at the surface to stabilize platinum NPs.^125^ Owing to its smaller particle size, the prepared composite outperformed the commercial Pt/C electrocatalyst for the electrochemical oxidation of methanol. Carbonized electrospun lignin nanofibers have also been applied as conductive support for Pd NPs^126^ and Ag NPs that were used as electrocatalysts for the oxygen reduction reaction (ORR).^127^ Figure 5 gives an example of the morphology of lignin‐derived electrospun nanofibers containing Ag NPs. Although the lignin–Ag NP composite electrocatalyst did not achieve the high mass activities of Pt_3_Ni nanoframe catalysts by far,^128^ it could still outperform a commercial Pt/C catalyst. Further, nitrogen‐doped carbon obtained from calcination of hydrothermally extracted and nitrated lignin has shown high electrocatalytic activity for ORR, comparable to those of non‐noble‐metal catalysts.^129^

Solid‐state photocatalysts are also regarded as heterogeneous catalysts, and lignin‐derived carbons can be coupled to semiconductors to yield composite photocatalysts.^130^, ^131^, ^132^ Khan et al. reviewed recently these carbon‐based composites for photovoltaic and photocatalytic applications.^133^ However, there are only a few reports focusing on photochemical applications of composites consisting of pristine lignin. This is mainly due to the unstable nature of lignin in contact with a medium, such as water, that can generate reactive radicals on light absorption by the dispersed semiconductor material. For example, rare‐earth‐doped TiO_2_ nanostructures can photodegrade lignin rapidly under simulated sunlight in water.^134^ Growing directly semiconductor nanostructures on lignin substrates may thus enable maintaining the metastable structure of lignin under operating conditions, similar to protective TiO_2_ coatings on oxynitride structures.^135^ Contrary to photocatalysis, lignin coatings can be used to quench the photocatalytic activity of semiconductors. For instance, lignin‐coated TiO_2_ NPs showed a decrease in possible phototoxicity, in the form of reactive radicals in sunscreen applications, due to the dissipation of photogenerated electrons in the lignin layer.^136^ More recently, ester‐linked lignosulfonate–TiO_2_ nanocomposite particles and admixtures of LNPs with ZnO NPs have been demonstrated for photoprotection applications.^137^, ^138^

### 2.6 Energy storage: components for batteries and supercapacitors

The constantly increasing demand for energy storage in batteries requires the development of new materials such as active electrode materials, current collectors, electrolytes, and separators. For lithium‐ion battery technology, organic electrolytes show high ionic conductivity and enable high energy density, but represent at the same time risks concerning leakage and flammability. The safety issues can be resolved by gel polymer electrolytes (GPEs) consisting of lithium, a plasticizer, and a polymeric matrix.^139^ However, the polymeric constituents such as poly(vinylidene fluoride) or poly(methyl methacrylate) are not biodegradable. The lignin–electrolyte interface enables metal‐ion exchange between two electrodes that make up the corresponding battery.

#### 2.6.1 Electrolytes and separators

Composites of lignin and polyvinylpyrrolidone synthesized by coupling by means of γ‐aminopropyltriethoxysilane have shown a high ionic conductivity of 2.52×10^−3^ S cm^−1^ as a lignin‐based GPE.^139^ Nanofibers produced from alkali lignin and poly(vinyl alcohol) and membranes obtained from cross‐polymerized lignin have been applied as porous separators for Li‐ion batteries.^140^, ^141^ The interface between lignin and inorganic charge carriers opens up applications of lignin–inorganic composite membranes also for Li–S^142^ and Zn‐ion^143^ batteries. Zn‐ion batteries show high theoretical capacity (820 mAh g^−1^), low electrochemical potential (−0.76 V vs. standard hydrogen electrode), operability in aqueous electrolytes, and high abundance. Membranes consisting of acid‐treated kraft lignin and Nafion can protect the metal surface, while the presence of the lignin–Zn^2+^ interface promotes the growth of lateral zinc hydroxide layer, that is, the effective solid‐electrolyte interphase (SEI).

Lignosulfonate has also been employed directly as electrolyte in a flow battery.^144^ The lignosulfonate undergoes a reversible redox reaction from phenolic hydroxyl to quinone. When paired with the Br_2_/Br^−^ redox couple, the full cell runs efficiently with high power density and achieves current densities of up to 20 mA cm^−2^. The unique complex molecular structure of lignin reduces the electrolytic crossover phenomenon, and thus ensures high capacity retention.

#### 2.6.2 Current collectors

Lignin is also a promising starting substrate for the synthesis of carbonaceous materials for energy storage.^145^, ^146^, ^147^, ^148^, ^149^, ^150^, ^151^, ^152^ The morphology and/or type of lignin as substrate has generally an influence on the properties of the final product, even if the conversion is performed at high temperatures above 723 K.^148^, ^153^, ^154^, ^155^, ^156^, ^157^, ^158^, ^159^ The electrochemical performance and stability of carbon‐based materials, such as graphite or hard carbon, is mainly determined by the SEI,^160^ that is, the decomposition layer separating the electrode from the liquid electrolyte that forms during first cycling. This general problem accounts for several electrode materials having a high theoretical capacity, such as Li_7_CuSi_2_ with 1563 mA h g^−1^,^161^ but continuous SEI formation preventing their application.

**Figure 6:**
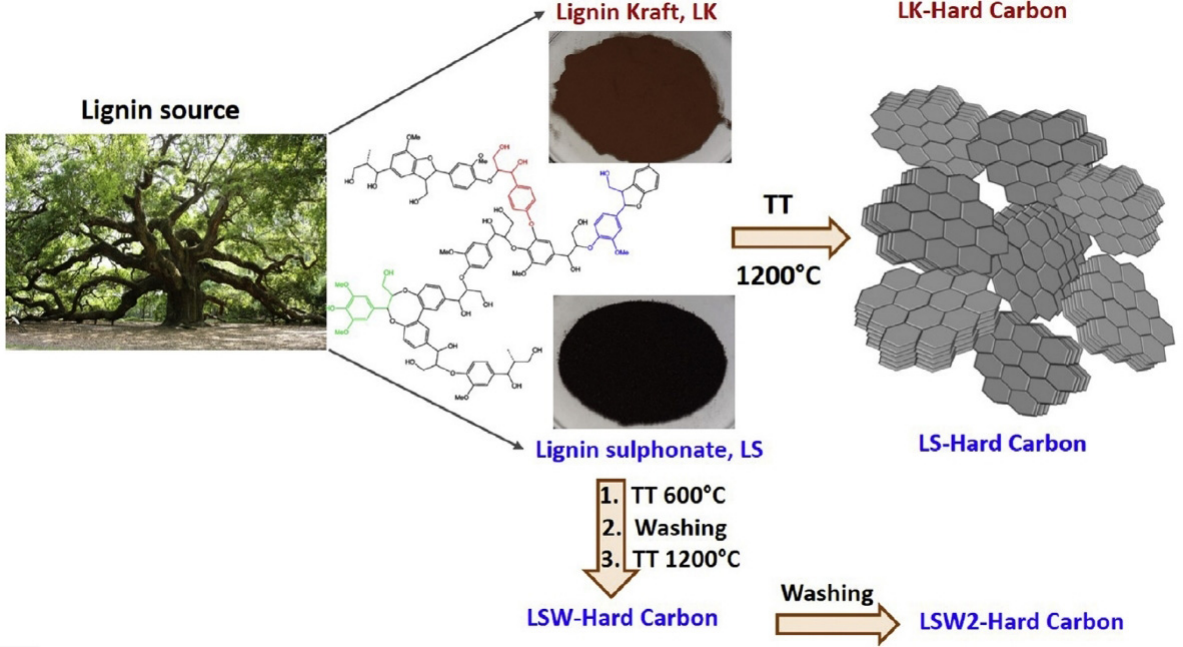
Synthesis of hard carbon derived from kraft lignin and lignosulfonate. Reproduced with permission from Ref. 158; Copyright Elsevier.

Ghimbeu et al. prepared hard carbon electrodes from kraft lignin and lignosulfonate that achieved steady capacities of 181 and 205 mA h g^−1^, respectively, versus Na^+^ (Figure 6).^158^ The impurity‐driven growth of the SEI resulted in significant capacity fading, but the authors could improve the stability, that is, continuous SEI formation, by applying washing steps to remove the impurities. These results indicate that lignin‐derived carbons can be competitive with other carbon materials for Na‐ion batteries, such as reduced graphene oxide electrodes (ca. 284 mAh g^−1^)^161^, ^162^

#### 2.6.3 Active electrode materials

The stability of a battery expressed as the Coulombic efficiency (CE), which is defined as the ratio between the charge delivered during discharge to the charge stored during the previous recharge, would reach a value of 100 % if no side reactions or degradation occurred in the battery.^163^ The CE should exceed at least 98 % for a material to be considered for implementation in commercial batteries. Furthermore, despite many encouraging results for lignin‐based separators and electrolyte components, the chemical stability of lignin in contact with the electrolyte or its additives should be an important criterion in its assessment as a “green” constituent of a battery.

**Figure 7:**
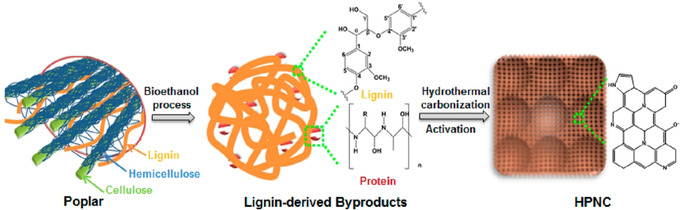
Diagram of the process for fabrication of interconnected hierarchical porous nitrogen‐doped carbon. Reprinted with permission from Ref. 166; Copyright American Chemical Society.

Carbonization can enable utilization of heterogeneous lignocellulosic biomass that is difficult to process by other techniques. For example, bioethanol production generates heterogeneous lignin‐enriched byproducts containing proteins and polysaccharides, which pose challenges for subsequent materials valorization or catalytic fractionation to chemicals.^164^ Hydrothermal carbonization of these byproducts resulted in interconnected hierarchically porous nitrogen‐doped carbon with a high specific area of 2218 m^2^ g^−1^ and a nitrogen doping content of 3.4 % (Figure 7). Supercapacitors based on this bioderived carbon exhibited a high specific capacitance of 312 F g^−1^ under alkaline conditions, which is less than that of 400 F g^−1^ reported for a bioderived carbon,^165^ but still among the highest values for state‐of‐the‐art carbon materials.^166^

**Figure 8:**
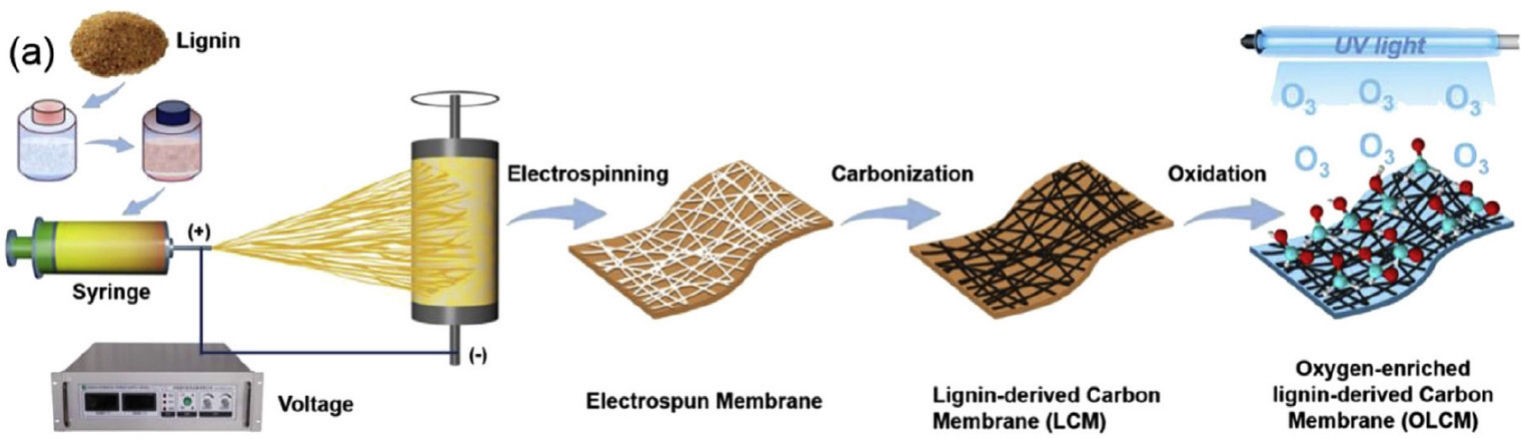
Diagram of the fabrication process of the lignin‐derived carbon membrane and corresponding oxygen‐enriched membrane. Reproduced with permission from Ref. 167, Copyright Elsevier.

A lithiophilic skeleton obtained by carbonization of electrospun fibers and subsequent ozone treatment (Figure 8) has oxygen‐rich surface functional groups. This improves the stability of batteries owing to uniform distribution of Li nucleation sites. A composite lithium‐metal anode based on lignin‐derived carbon achieved a coulombic efficiency of 98 % over 230 cycles.^167^ This approach has proven also successful for electrospun kraft lignin and cellulose acetate blended nanofibers to obtain carbons with a surface area of 541 m^2^ g^−1^. These porous carbon electrodes yielded a reversible capacity of 340 mAh^−1^ g^−1^ for Na^+^ intercalation,^168^ higher than that of 254 mAh^−1^ g^−1^ obtained with nanostructured carbon produced from lignin‐stabilized pitch emulsion.^169^ The carbon precursor and the carbonization process affect properties of the carbonaceous materials. In contrast to porous carbons obtained from electrospun nanofibers, lignin‐derived pyrolysis carbons were essentially nonporous, whereas porous carbons were obtained from cellulose and hemicellulose precursors.^170^ A possible reason for this difference is that fibrous components (mainly cellulose) provide structural support that avoids formation of dense lignin deposits during the carbonization process.

### 2.7 Other applications

There are a few notable lignin‐containing inorganic materials with intended use in applications other than those discussed above. Examples include enzyme immobilization for adhesive materials^171^ and reusable biocatalysts,^172^ as well as UV‐protective properties in polyurethane coatings.^173^, ^174^, ^175^ Another prospective application of lignin in which the functionality derives from its interface with an inorganic counterpart are environmentally friendly lubricants. Zhu et al. investigated lignin as an additive for choline–amino acid ionic liquids as lubricants. The addition of lignin enhanced the thermal stability and improved the antiwear properties and friction stability of steel.^176^ The lubricating properties were further improved by introducing nitrogen and phosphorus elements into the lignin structure. This resulted in augmented hydrogen‐bonding density between the additive and ionic liquids, which led to stronger adhesion of the lubricating layer to the metal surface.^177^ Lubricants based on ethylene glycol and poly(ethylene glycol) with lignin as additive have also shown anticorrosive characteristics on aluminum and iron.^178^

## 3 Outlook

The last few years have seen rapid advancements in lignin–inorganic materials. Lignin producers are also investing in piloting activities, such as carbonization of kraft lignin for energy storage applications. We foresee increasing political support for lignin‐based materials as enablers of circular economy. An example of this augmented awareness is the critical assessment of current technologies in energy storage. For the consideration of batteries as a green technology for energy storage, recycling of the components has to be implemented into the life‐cycle analysis of a manufactured battery. The disposal of used batteries to the environment combined with their nondegradability of components puts into doubt whether battery technology is currently truly sustainable. Lignin as a renewable and biodegradable feedstock for functional materials offers the opportunity to give established technologies a green footprint.

However, more efforts are needed to develop separation processes that enable recycling of the inorganic components such as metal ions. We believe that lignin has the potential to be one of the key compounds that can replace current synthetic polymers and carbons that are sourced from fossil oil and gas. In particular, we foresee that lignin can make an impact in: 1) Composite adsorbent materials for recovery of metals and purification of water, in which precise control over the adsorption selectivity may trigger also the design of hierarchical structures for photocatalytic systems; 2) Batteries and supercapacitors free of fossil carbon; 3) Lignin‐supported heterogeneous catalysts in which the lignin–metal interface plays an active part in the catalyzed reaction; 4) Lignin‐based materials for carbon capture and storage; and 5) Particulate coatings that benefit from the multifunctional properties of lignin.

Technical lignins are severely modified during the pulping process and differ markedly from lignins isolated by using alternative solvents and methods, for example, “lignin‐first” approaches. Future work should better describe the origin and properties of the lignin starting materials to help identify the most suitable lignin fractions, physicochemical modification routes, and ultimately a structure–function relationship in applications. Achieving this goal will also help to decrease the current dependence on the pulp and paper industry as the predominant source of lignin. Future work should also aim to scale up synthesis and demonstrate the materials under real application conditions.

## Conflict of interest

*The authors declare no conflict of interest*.

## Biographical Information

Tetyana M. Budnyak was born in 1986 in Ukraine. In September 2016 she obtained a PhD degree in Physical Chemistry at the Chuiko Institute of Surface Chemistry, Ukraine (Prof. Valentin Tertykh) after completing joint research at Maria Curie‐Sklodowska University, Poland (Prof. Dorota Kolodynska). She moved in 2017 to the KTH Royal Institute of Technology, Sweden for postdoctoral work at the group of Prof. Mikael Lindström. In May 2019 she joined the group of Ass. Prof. Adam Slabon at the Department of Materials and Environmental Chemistry (MMK) at Stockholm University, Sweden. During her postdoctoral studies she has conducted research stays at Yale University, CT, USA (Prof. Julie Zimmerman and Prof. Paul Anastas). Her main research interests are devoted to the development of sustainable functional materials for environmental applications.

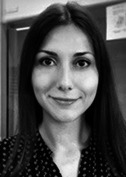

## Biographical Information

Adam Slabon was born on November 24th 1983 in Hindenburg, Poland, and grew up in Nuremberg, Germany. He graduated in chemistry at the Jagiellonian University in Cracow, with research visits to NTU in Singapore and EPF Lausanne in Switzerland. He obtained his PhD degree from ETH Zurich (Prof. Reinhard Nesper) in 2013 and moved to the University of California, Berkeley, for postdoctoral studies. In 2014, he initiated his independent research at the Institute of Inorganic Chemistry, RWTH Aachen University, Germany, where he obtained his habilitation (Mentor: Prof. Richard Dronskowski) in 2019. In the same year, he joined MMK at Stockholm University, Sweden, as Assistant Professor (tenure‐track). His research interests are centered at the development of materials and sustainable methods for environmental applications and solar energy conversion.

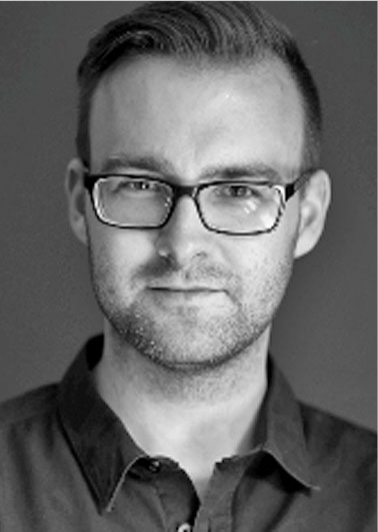

## Biographical Information

Mika H. Sipponen, born 1983 in Finland, received his D.Sc. (Tech.) degree in the field of chemical technology from Aalto University in 2015 (Prof. Simo Laakso). He has worked on lignin‐related topics since 2008, from biorefining of straw to lignin nanomaterials. From January 2016, he worked as a Research scientist in VTT prior to receiving a postdoctoral research grant from Academy of Finland. As a post‐doctoral researcher, he worked on lignin‐based functional materials with Prof. Monika Österberg. His research stays and short visits have taken place in China (Prof. S. Deng, 2009), France (Prof. S. Baumberger, 2012), Italy (Prof. C. Crestini, 2017), and Japan (Prof. Y. Amano, 2019). Since September 2019, he is an Assistant Professor in Materials chemistry in MMK at Stockholm University. His Sustainable materials chemistry group develops lignin‐based materials mainly for energy and environmental applications.

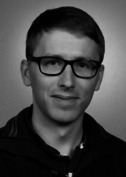

## Supporting information

As a service to our authors and readers, this journal provides supporting information supplied by the authors. Such materials are peer reviewed and may be re‐organized for online delivery, but are not copy‐edited or typeset. Technical support issues arising from supporting information (other than missing files) should be addressed to the authors.

Supplementary
