# Supplementary material for: Lignin–Inorganic Interfaces: Chemistry and Applications from Adsorbents to Catalysts and Energy Storage Materials
Source: ChemSusChem. 2020 Apr 17;13(17):4344–55. doi: 10.1002/cssc.202000216 (PMC7540583; doi:10.1002/cssc.202000216)
Supplement: Supplementary file 1 — Supplementary [file CSSC-13-4344-s001.pdf]

Author Contributions

T.B. Conceptualization: Equal; Visualization: Lead; Writing - Original Draft: Lead; Writing - Review & Editing: Equal
A.S. Conceptualization: Equal; Funding acquisition: Equal; Resources: Lead; Visualization: Lead; Writing - Original Draft: Equal; Writing - Review & Editing: Equal
M.S. Conceptualization: Lead; Funding acquisition: Supporting; Methodology: Equal; Project administration: Equal; Resources: Equal; Supervision: Lead; Visualization: Equal; Writing - Original Draft: Equal; Writing - Review & Editing: Lead.
